# Beyond the Syndrome: Extensive Congenital Abnormalities in an Infant With Trisomy 21

**DOI:** 10.1177/2632010X221088966

**Published:** 2022-04-22

**Authors:** Jeremy D Ward, Mahesh S Sharma, Matthew F Pizzuto, Vincent J Moylan, Frederic B Askin, David G Kaufman

**Affiliations:** 1 Department of Pathology and Laboratory Medicine, University of North Carolina School of Medicine, Chapel Hill, NC, USA; 2 Department of Surgery, University of North Carolina School of Medicine, Chapel Hill, NC, USA; 3 Department of Pediatrics, University of North Carolina School of Medicine, Chapel Hill, NC, USA

**Keywords:** Trisomy 21, Down syndrome, atrioventricular septal defect, tetralogy of Fallot, necrotizing enterocolitis, abnormal intestinal villi formation, abnormal tracheal bronchus, bowel adhesions

## Abstract

Herein we discuss the clinical course and subsequent autopsy of a female infant with trisomy 21 with balanced Rastelli Type “C” complete atrioventricular septal defect (AVSD), tetralogy of Fallot and right aortic arch with mirror image branching pattern who underwent a palliative right modified Blalock-Taussig-Thomas shunt (mBTTS) for hypoxemia from progressive right ventricular outflow tract obstruction. The baby was found to have multiple concomitant pathologic findings not typically seen with this constellation of cardiac anatomy. Autopsy revealed significant abdominal adhesions with near-complete stenosis of the transverse colon. In addition, the infant was found to have significantly elongated villi within the small and large bowel and a relatively large collagenous polyp in the small bowel. The decedent also had an abnormal tracheal bronchus, characterized by an additional superior right-sided bronchus, which is an extremely rare abnormality. Her clinical course was complicated by severe pulmonary hypertensive arteriolar changes out of proportion to what would be typical for her age, trisomy 21 status, and degree of left to right intracardiac shunting. Furthermore, she had refractory anasarca and recurrent chylous pleural effusions without gross lymphatic abnormalities that may have been secondary to systemic capillary leak syndrome (SCLS) versus severe pulmonary hypertension. Due to the aforementioned findings, the family elected for comfort care and the baby expired shortly after extubation. Overall, the infant had multiple, rare coexisting congenital abnormalities that likely represents an extreme phenotype of trisomy 21 that has not been described in the literature to date.

## Introduction

Approximately 45% to 50% of trisomy 21 patients are affected by congenital heart defects, with approximately 40% of these affected patients having an atrioventricular septal defect (ASVD).^1,2^ Twice as many females with trisomy 21 are affected with AVSD than males. Conversely, tetralogy of Fallot (TOF) is reported to be present in approximately 2% to 6% of trisomy 21 patients^2,3^ and, thus, is relatively uncommon in patients with trisomy 21. Consequently, the presence of both AVSD and TOF together in an individual, even one with trisomy 21, is rare. A review of the literature found that ASVD is associated with TOF in 5% to 10% of patients with AVSD and in 1.7% of patients with TOF.^4^ Given the rarity of such patients, how these malformations affect underlying physiology is not fully understood nor reported in the literature. Therefore, in this report, we discuss the clinical course and the autopsy findings of an infant with trisomy 21 who had multiple congenital abnormalities that likely represents the extreme of the spectrum of possible concomitant abnormalities in patients with trisomy 21.

### Clinical history

The patient was a 12 weeks and 3 days old female that was born at 38 weeks and 4 days to a 37-year-old G6P3 mother. The patient was diagnosed prenatally with trisomy 21, complete balanced atrioventricular septal defect, and tetralogy of Fallot (AVSD/TOF) defects of the heart. After an uncomplicated spontaneous vaginal delivery, the neonate was admitted to the intensive care unit. On day 2 of life, the baby developed necrotizing enterocolitis (NEC). She was treated with bowel rest and intravenous antibiotics. Enteral feeds were subsequently resumed. Shortly thereafter, the decedent was noted to have a decrease in oxygen saturation to lower 80’s percent oxygen saturation from a previous baseline of mid-90s percent oxygen saturation. Gastro-esophageal reflux disease and significant trachea-bronchomalacia was ruled out as a cause of the desaturation. An echocardiogram demonstrated an increase in dynamic right ventricular outflow tract obstruction (RVOTO) with a small patent ductus arteriosus. Treatment was initiated with beta blockade; however, she remained symptomatic and repeat cardiac ultrasound demonstrated severe valvar and sub-valvar right ventricular outflow tract obstruction and minimal ductal blood flow to the pulmonary arteries. A prostaglandin infusion was started to maintain duct patency. Due to the progressive RVOTO and lack of favorable anatomic features for an interventional catheterization approach, a surgical systemic to pulmonary artery shunt procedure was recommended.

The infant underwent a successful placement of a mBTTS from the right branchiocephalic artery to right-main pulmonary artery confluence using a 3.5 mm ePTFE graft and was transferred to the pediatric cardiac intensive care unit (PCICU) after her operation on a heparin infusion. Several hours later, the child suffered a bradycardic arrest. Open chest resuscitation was performed, and inspection of the shunt revealed antegrade perfusion from the innominate artery with retrograde perfusion of the pulmonary arterial system without evidence of thrombosis. Echocardiogram revealed continuous flow in the shunt with laminar flow into both branch pulmonary arteries. As such, it was thought that the etiology was respiratory in nature as mucous plugs were evacuated from the airway during the event. The infant clinically improved with resuscitation and open chest physiology; and by visual inspection, her heart contractility improved markedly. Her chest was closed the following day and she was extubated a week later. However, a few days after extubation, the infant had worsening respiratory status and was intubated and placed on mechanical ventilation, on which she remained for the remainder of her hospital course.

The infant required multiple chest tubes for recurrent chylous and serous pleural effusions. Ultrasonography revealed no thrombus formation in the central venous system. Moreover, she had persistent hypoalbuminemia and gross anasarca of the chest and abdominal wall despite replacement therapies and aggressive diuresis. She experienced atrial tachyarrhythmias requiring anti-arrhythmics including amiodarone and procainamide. Additionally, the baby had a second episode of NEC on resumption of feeding with a medium chain triglyceride (MCT) diet. For this second episode of NEC, the infant completed a 14-day course of broad-spectrum antibiotics and bowel rest. Small volume feeds of skimmed maternal breastmilk were eventually restarted. However, due to lack of clinical improvement, the infant underwent cardiac catheterization that revealed a widely patent systemic to pulmonary artery connection.

The infant eventually developed severe anasarca that was refractory to medical treatment. Late in her hospital course, she returned to the cardiac catheterization lab to evaluate the mBTTS shunt for possible stenosis or occlusion. The catheterization revealed a patent mBTTS shunt, no pulmonary artery stenosis, and the presence of severe pulmonary hypertension. After a multi-disciplinary discussion with the medical teams including critical care, cardiology, palliative care, and cardiothoracic surgery, the family decided to re-direct care toward comfort, given the infant’s constellation of problems and limited treatment options. The infant was made do-not-resuscitate (DNR) status. The infant was then extubated at the family’s request. After extubation, the infant quickly progressed to hypoxemic respiratory failure leading to cardiac arrest and died.

### Autopsy findings

At postmortem examination, the decedent weighed 14.0 pounds, with a crown-rump length of 36.0 cm and a crown-to-heel length of 55.0 cm. The decedent had a head circumference of 37.5 cm, a chest circumference of 43.0 cm and an abdominal circumference or 44.6 cm. The head was noted to be moderately brachycephalic with both fontanels present and bulging. The decedent had facies of trisomy 21 that included epicanthic folds, up-slanting palpebral fissures, brachycephaly, flattened nose, and slightly low-set ears with somewhat dysmorphic folding. The upper lip had a marginally reduced philtrum, with a full vermillion border. The hard and soft palates were intact, and no teeth were present. There was no cleft lip or palate. The neck was symmetrical with no abnormal masses but was somewhat shortened.

The chest was normally formed and symmetrical with normally positioned nipples. A midline chest incision that measured 7.8 cm in length was present and closed with suture. The incision was well approximated, except for an area of breakdown in the inferior-most area near the xiphoid process. This area of breakdown in the incision measured 1.6 × 1.1 cm and had well-healing granulation tissue present. There was no erythema or crepitus surrounding the incision site.

The exterior anterior abdominal wall was intact. The abdomen was symmetrical but was significantly distended. The spine was straight and free of defects. The gluteal folds were normal and symmetrical. There was a 2.5 cm linear abrasion/skin breakdown to the dermis in the upper gluteal cleft. The external genitalia were those of a normal immature female. The labia were normally formed but very swollen and edematous. The vagina was patent. There were no inguinal hernias. The anus was patent and there was stool present within the rectum. All 4 extremities were formed normally with no contractures, webs or strictures. There was clinodactyly of the hands and sandal gap deformities of the feet present bilaterally. The fingers and toes were of grossly normal configuration. There was a small 0.3 cm abrasion on the dorsal surface of the right thumb. The fingernails of both hands were cyanotic. Conversely, the toenails were pink and not cyanotic.

**Figure 1.**
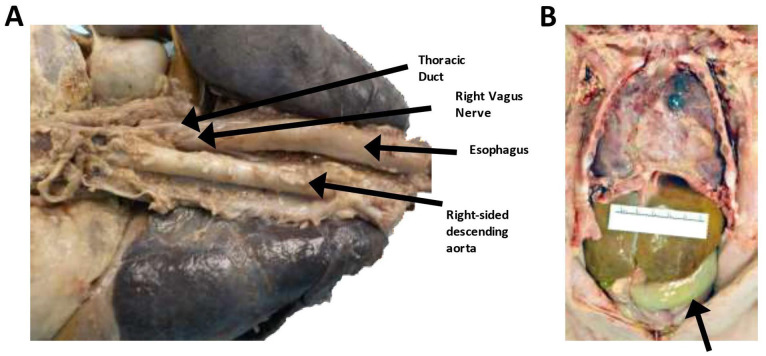
Gross internal photographs of the decedent demonstrating thoracic and abdominal adhesions and a dilated transverse colon. (A) Posterior view of the thoracic and upper abdominal structures demonstrating patent thoracic duct and right-sided aorta. (B) Internal organs of the chest and abdomen demonstrated significant adhesions and a distended transverse colon.

The esophagus was patent and emptied into the stomach. The esophagus traversed the thorax just left of midline and behind the trachea and left bronchus (Figure 1A). There were no fistulae or focal lesions. The stomach was increased in size but was normally formed and free of ulcers (Figure 1B). The pylorus was of normal structure and circumference, with no pyloric stenosis. There was approximately 20 mL of green, semisolid material within the stomach. The rugae were normal. The duodenum curved around the head of the pancreas and was patent. The jejunum, ileum and appendix were grossly normal. The colon was normally positioned. On microscopic evaluation, the sections of the stomach demonstrated normal architecture. The sampling of the pylorus demonstrated an appropriate muscular wall with normal appearing antral mucosa. Interestingly, the section of the duodenum demonstrated elongated villi with normal-appearing submucosa within the villi.

**Figure 2.**
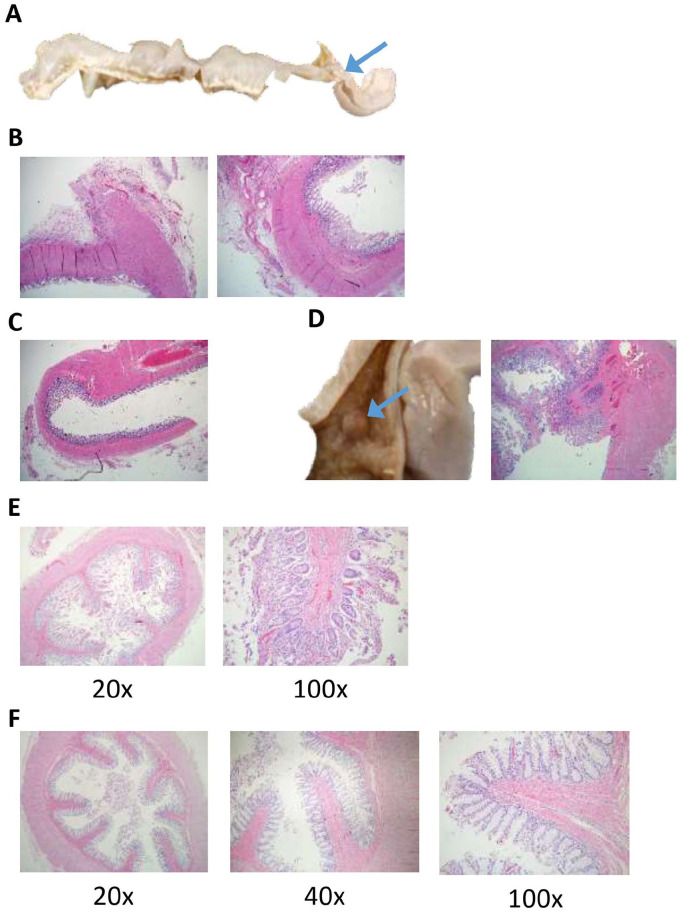
Adhesions and stenosis of the transverse colon with unusually enlarged villi were identified microscopically within the small and large bowel. (A) The transverse colon that has been removed and opened. The proximal portion of the transverse colon was significantly dilated and thinned with an area of stenosis (blue arrow) within the distal transverse colon. (B) H&E section of the grossly stenotic bowel demonstrated fibrotic adhesions on the antimesenteric side (left image) with scattered chronic inflammatory cells permeating through the fibrotic areas around the serosa. The muscular wall was significantly thickened (right image). The mucosa was roughly intact with minimal autolysis. (H&E, 40×) (C). H&E section of the transverse colon just proximal to the area of stenosis. It was relatively well-preserved with mucosa demonstrating eosinophilia on the distal aspect of the mucosa suggestive, but not definitive for ischemia versus autolysis. The muscular wall was significantly thinned, especially compared to the adjacent grossly stenosed bowel (seen in part B) (H&E, 40×). (D) Photograph of a single polyp (blue arrow) that was identified within the small intestine (left). An H&E section of the polyp (right) demonstrates that the polyp had a stalk composed of normal appearing muscularis tissue that was covered with normal small intestinal mucosa. There was an abnormal amount of submucosa and possibly muscularis associated with the polyp. Adjacent to the polyp there are distinct, elongated villi were present. (H&E, 40×) (E). The representative samples of the small intestine demonstrated significantly elongated villi with minimal autolysis. (H&E, 20× and 100×; as labeled) (F) The rectal tissue demonstrated long villi that are not typically seen in the rectum with well-preserved rectal mucosa, patent, with bits of stool. (H&E, 20×, 40×, and 100×; as labeled).

The ascending and the transverse colon were significantly enlarged and distended (Figure 1B). There were no volvuli or malrotations identified; however, there were significant abdominal adhesions, with multiple areas of the small bowel adhered to the overlying abdominal wall. An area of non-occlusive stenosis was identified within the distal transverse colon (Figure 2A). Grossly, the mucosa and wall of the transverse colon, before the area of the stenosis, was significantly thinned. The contents of the small intestine and colon were bile stained. There was no evidence of mucosal air or pseudomembrane formation.

On microscopic evaluation, the grossly stenotic large bowel had fibrotic adhesions on the antimesenteric side with scattered chronic inflammatory cells permeating through the fibrotic areas around the serosa. The muscular wall was significantly thickened in the area just before and after the area of stenosis (Figure 2B). Conversely, the distended and thin transverse colon proximal to the area of stenosis (Figure 2C) was relatively well preserved with mucosa demonstrating eosinophilia on the distal aspect of the mucosa suggestive, but not definitive, for ischemia. The muscular wall was significantly thinned, especially compared to the adjacent grossly stenosed bowel (Figure 2B vs Figure 2C). Additionally, a small intestinal polyp (2 mm) was identified. The polyp had a small stalk and was composed of normal appearing muscularis tissue that was covered with normal small intestinal mucosa (Figure 2D). Curiously, there was an abnormal amount of mucosa and muscularis associated with the polyp. The villi were abnormally elongated throughout the entire small bowel. The appendix was present and measured 5.2 cm in length. Microscopically, the appendix appeared normal. A sample of the rectum also demonstrated significantly elongated villi (Figure 2E). Neural tissue with ganglia were identified surrounding the rectum. Overall, the small and large bowel tissue displayed very long and prominent villi that may represent part of the abnormal phenotype seen in the decedent, which has not been described in the literature.

The liver was normally formed and there were no focal lesions. The liver weighed 223 g (expected 149 g [fifth percentile] to 254 g [95th percentile]). The gallbladder measured 3.7 × 1.5 cm, was normally formed, and not distended. Surprisingly, pressure on the gallbladder did not cause expression of bile from the ampulla of Vater into the duodenum. Similarly, the biliary tract, from the ampulla of Vater, could not be traversed with a small metal probe. However, there was a small, palpable firm swelling beneath the ampulla of Vater and the associated pancreas. On cut section, this area was much darker in color compared to the body of the pancreas, indicative of necrosis. The pancreas had a normal anatomic location and configuration and there were no cysts or other focal lesions identified. There was a single spleen with no focal lesions present. The spleen weighed 14 g (expected 7 g [fifth percentile] to 29 g [95th percentile]).

On microscopic evaluation, the sections of the liver demonstrated normal liver parenchyma. There was minor, focal biliary congestion that appeared acute. There was no vascular congestion. The portal triads were intact and there was no indication of thrombosis or inflammation. The pancreas was well preserved and had a normal architecture with acinar and islet cells, appropriate for gestational age. However, directly below and adjacent to the ampulla of Vater there was significant necrosis of the pancreas. This completely necrotic area transitioned into pancreatic acini in the process of undergoing necrosis with significant macrophage infiltration. The pancreatic and biliary duct were both visualized on microscopic sectioning and were fully necrotic. The outpouching of the ampulla of Vater was visualized and the lumen appeared patent. A section of the spleen demonstrated extensive vascular congestion but was otherwise normal.

The right pleura and lung tissue were extensively adherent to the anterior chest wall (Figure 1B). There were widespread adhesions throughout the right pleural cavity, with most of the pleural surface adherent to the right chest wall. The left pleura had multiple scattered adhesions, especially to the lateral chest wall and over the anterior and posterior surfaces. There was no evidence of pneumothorax or mediastinal emphysema. There was minimal serosanguinous fluid in the right and left pleural cavities. The mediastinum had multiple fibrous adhesions to the rib cage and to the tissue over the area of the heart. The pericardial sack was surgically resected from over the entire anterior surface of the heat and was present only over the lateral-most and inferior borders of the heart (Figure 1B). There was no fluid within the pericardium.

**Figure 3.**
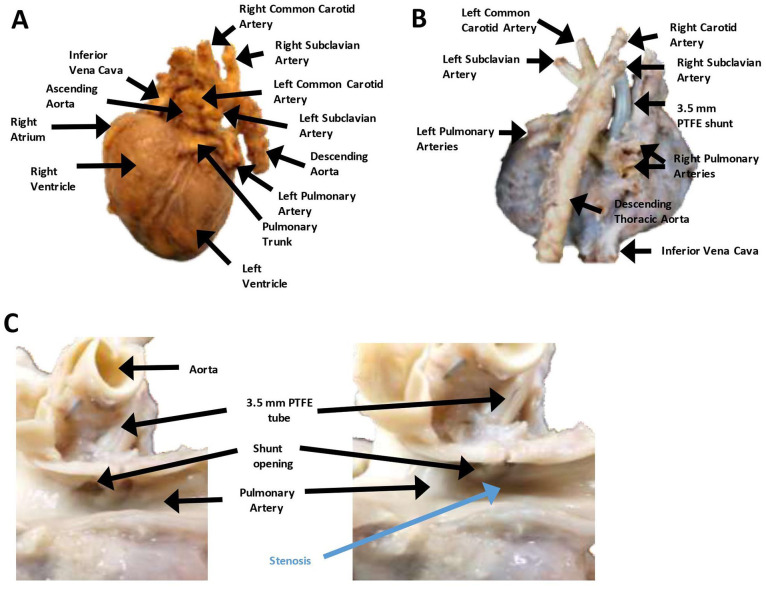
The decedent’s heart demonstrating a right-sided aorta with mirror image branching, patent mBTT-shunt and significant pulmonary artery stenosis immediately distal to the shunt. (A) Anterior view of the decedent’s heart demonstrating a right-sided aorta and mirror image branching of the arteries. All major structures are labeled. (B) Posterior view of the decedent’s heart demonstrating a right-sided aorta and mirror image branching. The 3.5 mm PTFE shunt is clearly visible. All major structures are labeled. (C) Close up view of the pulmonary artery after longitudinal sectioning of the artery and opening of the PTFE shunt. There was a patent shunt with no thrombosis or stenosis at the distal end of the shunt (left); however, there is significant stenosis of the pulmonary artery that is just distal to the distal opening of the PTFE shunt (right; labeled in blue). All major structures are labeled.

The ascending aorta was noted to be right-sided, rather than in its normal left-sided orientation (Figures 1A, 3A, and B). There was mirror image branching, with the left carotid and left subclavian arteries arising from a small left brachiocephalic artery (Figure 3A and B). The left subclavian artery passed in front of and to the left of the trachea (right aortic arch and left ductus arteriosus; anterior type). The right subclavian and right carotid artery arose independently from the aorta (Figure 3A and B). A 3.5 mm polytetrafluoroethylene (PTFE) modified Blalock-Taussig-Thomas shunt (mBTTS) was noted to run from the right carotid artery to the right pulmonary artery (Figure 3B and C). The mBTT shunt anastomoses were intact with no indication of leak. The mBTT shunt was patent with no indication of stenosis or clot. However, just distal to the mBTT shunt was an area of significant stenosis within the pulmonary artery (Figure 3C). The ductus arteriosus was noted to be successfully ligated, with no indication of leak. The aorta had no thrombosis or stenosis. The celiac artery, superior mesenteric artery, inferior mesenteric artery, and bilateral renal arteries were patent and had no thrombosis present.

**Figure 4.**
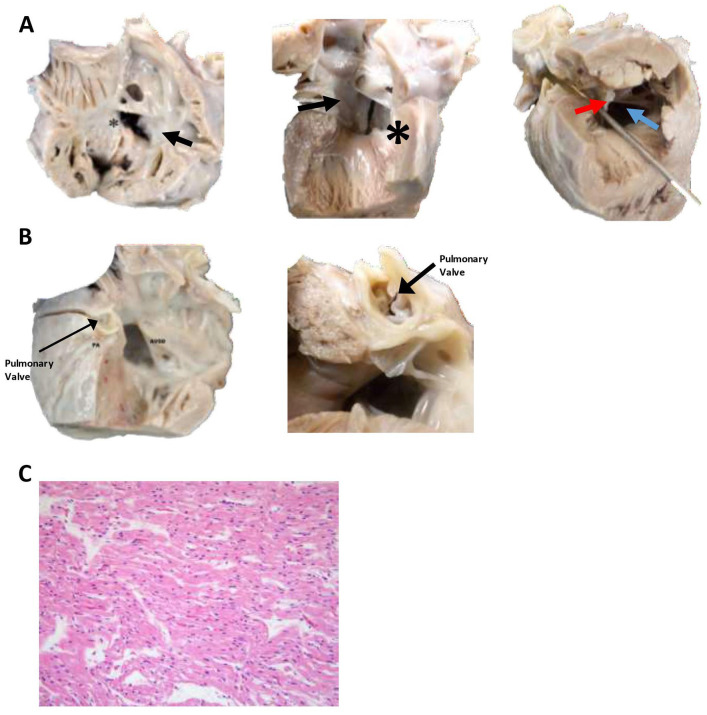
Sectioning of the heart demonstrates a large atrial-ventricular defect and pulmonary valve that was significantly decreased in size. (A) Multiple views of the Rastelli Type C atrial-ventricular septal defect. The picture on the left is of the opened right atrium and right ventricle demonstrating an atrial-ventricular septal defect (*). The middle picture is of the interior of the left atrium and left ventricle demonstrating the other side of the atrial-ventricular septal defect (*). Both views demonstrate the tricuspid valve connecting with mitral valve through the ventral defect and attaching to the anterior papillary muscle of the right ventricle (Rastelli Type C; arrow). Finally, the picture on the far right is looking up through the left ventricle demonstrating the atrial-ventricular septal defect (blue arrow) and the tricuspid valve connecting with mitral valve through the ventral defect (red arrow), crossing through the path of the aortic outflow tract (metal probe is traversing the aortic outflow tract). (B) Opened left aorta and ventricle demonstrating atrial-ventricular septal defect (ASVD) and the pulmonary valve within the pulmonary artery (PA). The picture on the right is heart rotated 90 degrees and showing the small (2 mm) pulmonary valve. (C) A representative section of the left ventricle demonstrates elongated myocytes with eosinophilic cytoplasm, appropriate for gestational age. There was minimal vascular congestion of the tissues. No leukocyte infiltrate or coagulative necrosis was identified. Overall, no microscopic abnormalities were identified. (H&E, 400×.).

The heart weighed 49 g and was larger than normal for the decedent’s weight and age (Figure 3A and B; expected weight, 21-35 g). The heart had extensive fibrosis over the anterior surface. The right ventricle was anterior, and the pulmonary trunk was normally positioned. The myocardium was red and moderately firm without focal lesions. The right and left atrial appendages were in their normal positions. The right atrium was increased in size. The superior vena cava and the inferior vena cava were on the right and both entered the right atrium, as did the coronary sinus. There was a complete common atrioventricular septal defect. This was classified as a Rastelli classification, type “C” by transesophageal and transthoracic echocardiography, which was confirmed at autopsy. Accordingly, there was a free-floating common atrioventricular valve anterior leaflet without chordal attachment to interventricular septum crest. The superior (anterior) leaflet of the tricuspid was undivided and did not attach to the interventricular septum. Instead, it attached to the anterior papillary muscle of the right ventricle. There was a moderate-size atrial component and a large ventricular component of the atrioventricular canal defect (Figure 4A). The aorta overrode the ventricular septal defect. There was also an ostium primum defect, with a defect in the inferior portion part of the atrial septum at the level of the atrioventricular valves. The foramen ovale was absent (destroyed by the atrial defect). The leaflets of the tricuspid valve were thin, pliable, and translucent. The right ventricular chamber was somewhat globular and was lined by thin endocardium. The right ventricle was mildly hypertrophied. The pulmonary outflow tract was very stenotic (Figure 4B). There was slight anterior deviation of the outlet septum. The pulmonic valve was normal in shape but exceedingly small (2 mm; Figure 4B). The pulmonic valve leaflets were thin, semitransparent and pliable. The pulmonic trunk gave rise to the right and left pulmonary arteries. The patent ductus arteriosus had been surgically removed. The histologic appearance of the heart was normal with no indication of infarct or necrosis (Figure 4C).

**Figure 5.**
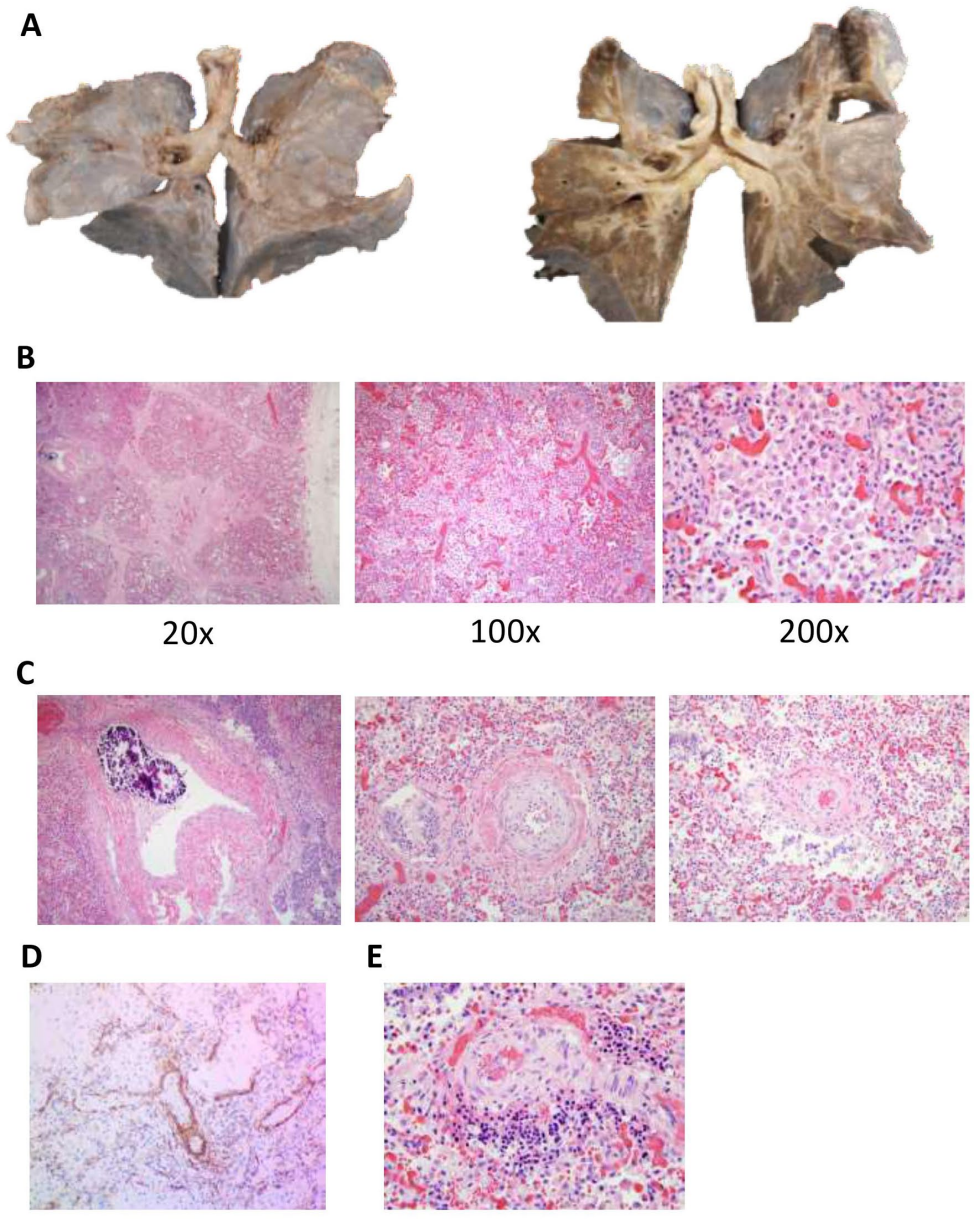
Abnormally shaped right tracheal bronchus with heavy, immature, edematous lungs, and severe pulmonary stenosis. (A) Photographs of the anterior surface of the decedent’s lungs both uncut (left) and coronally sectioned (right). There was an abnormally shaped right bronchus, with a large upper right bronchus going into the upper lobe and a second large lower right bronchus going into the middle lobe that has a branch within the middle lobe supplying right lower lobe. The left bronchus was singular and of normal configuration. The right lung has 3 lobes. The left lung has 2 lobes. The pleura and parenchyma of both lungs were roughened and somewhat fibrotic. The pulmonary arteries and veins were patent and have a grossly normal configuration. On cut surface, the lungs were firm and not well aerated. (B) Representative H&E stained lung tissue at indicated magnifications. At 20×, there was increased fibrin deposition on the surface of the lung consistent with the significant adhesions seen grossly. At 100× and 200×, most of the lung parenchyma was nearly filled with macrophages (many of which are hemosiderin-laden), indicative of severe congestion and significantly decreased elasticity of the lung. There was moderate vascular congestion as well, with both nucleated and non-nucleated RBCs visualized. There was significant loss of the overall structure of the alveoli with very immature alveoli seen in areas with less infiltration of macrophages and RBCs. (H&E, 20×, 100×, 200×; as labeled) (C) A representative H&E section demonstrating severe features of pulmonary hypertension, with severe calcification more than would be expected for the age of this patient, with marked medial thickening and intimal fibrosis. There was interstitial fibrosis, a feature of pediatric pulmonary hypertension, and seen in patients with hypoxia. There was extensive calcification of the vessels, which is a secondary phenomenon related to arterial damage and/or metabolic derangements. (H&E,100×). (D) The lung tissue was stained with smooth muscle actin and demonstrates there is no extension the smooth muscle layer into the distal acinar vessel (the normally non-muscularized intra-acinar arteries) indicating there was no medial hypertrophy. (E) This section of the lung demonstrates one of the focal areas of extramedullary hematopoiesis, which is indicative of profound hypoxia.

The larynx was formed normally, patent, and in a midline position. The combined lung weight was 85.2 g (expected weight, 80 g [fifth percentile] to 160 g [95th percentile]). The tracheobronchial tree was patent. There was an abnormally-shaped right bronchus, with a large bronchus going into the right upper lobe and a second large bronchus going into the right middle lobe that had a branch within the right middle lobe supplying the right lower lobe (Figure 5A). The left bronchus was singular and of normal configuration. The right lung had 3 lobes. The left lung had 2 lobes. The pleura and parenchyma of both lungs were roughened and somewhat fibrotic (right was worse than the left). As noted above, there was significant stenosis of the main pulmonary artery. The remaining portions of pulmonary arteries and veins were patent and had a grossly normal configuration. On cut surface, the lungs were firm, non-crepitant and not well aerated.

Clinically, the decedent had decreased thyroid hormone levels. However, on autopsy the thyroid gland was normally positioned, bilobed and symmetrical. The thyroid was of normal size and did not have any nodules or goiters visualized grossly. On microscopic evaluation, there were no anomalies noted. The adrenals were in their correct positions. The cortices of both glands were normally formed and were yellow in color. There was no evidence of hemorrhage or tumor. The combined adrenal gland weight was 3.1 g (expected 2.1 g [fifth percentile] to 6 g [95th percentile]). On microscopic evaluation, the adrenal glands were relatively well preserved with normal architecture, mild vascular congestion, and were appropriate for gestational age.

On microscopic examination analysis of the lung tissue, there was fibrin deposition on the surface of the lung, consistent with the significant adhesions seen grossly. Both the upper lobes and the right lower lobe, and to a lesser degree, the left lower lung parenchyma was nearly filled with macrophages (Figure 5B). The lung parenchyma was immature with very few mature alveoli present. The alveoli and alveolar ducts were dilated and there was a distinct double capillary layer in the interalveolar septa.^5^ The radial alveolar count was not diminished, which is not typically seen in neonates with trisomy 21.^5^ In addition, there were prominent intrapulmonary bronchopulmonary vascular anastomoses.^6^ There were distinct histologic changes related to pulmonary hypertension with marked thickening of the media of the muscular pulmonary arteries. The muscular pulmonary arteries also displayed intimal fibrosis with extensive calcification of the pulmonary vessels, typically a secondary phenomenon related to arterial damage and metabolic disorders (Figure 5C). However, there was no evidence of extension of a complete smooth muscle layer into distal acinar vessels (Figure 5D). There was also scattered, focal areas of extramedullary hematopoiesis, indicative of profound hypoxia (Figure 5E). No bacteria were visualized in the lung parenchyma or in the alveolar spaces and bacterial cultures of the lungs were negative.

**Figure 6.**
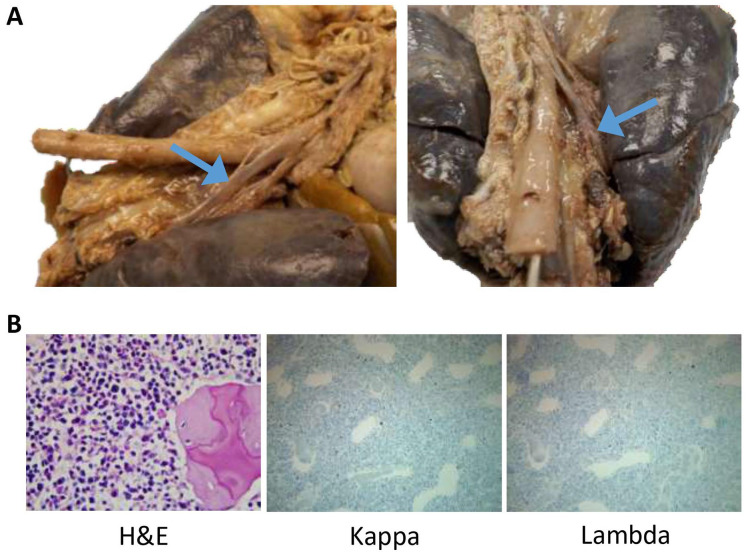
No anatomic abnormalities of the thoracic duct or hematologic malignancies were identified to account for the decedent’s chylothorax. (A) Additional views from the posterior surface of the thoracic cavity demonstrating an intact and patent thoracic duct (blue arrows). (B) The H&E section of the vertebral body demonstrated cartilage surrounding pink bone with bone marrow spaces that contain normal trilineage hematopoiesis occupying nearly 100% of the bone marrow. A Kappa and Lambda ISH stain were performed that showed appropriate 2:1 Kappa to Lambda staining, further ruling out the possibility of leukemia or lymphoma. (H&E 400×; Kappa and Lambda ISH, 200×).

A single thoracic duct arose from the cisterna chyli (Figure 6A). It traversed into the thoracic cavity and ran posterior to the esophagus. It then crossed from midline to just right of midline, at the level of approximately two-thirds of the length of the esophagus up to the junction of the first and second thirds of the esophagus (from the proximate end). Thereafter, it traversed left behind the trachea making its way superiorly and anteriorly. It eventually connected with the left subclavian vein. The main thoracic duct was patent and intact throughout entire length with no indication of injury or stenosis. Smaller tributaries were identified in the abdomen and lower thorax, which appeared to have no injury. However, small tributaries were not identified in the upper thorax.

Due to presence of chylothorax clinically, we further investigated other causes of chylothorax. Another cause of chylothorax in infants is leukemia and/or lymphoma.^7,8^ Infants with trisomy 21 are at increased risk for developing myeloid leukemia of Down syndrome (ML-DS) and acute lymphoblastic leukemia.^9^ Grossly, the vertebral and rib bone marrow were granular, red and homogenous with no indication of malignant cells. Microscopically, the rib demonstrated skeletal muscle adjacent to bone containing bone marrow spaces. The marrow was stained with PAS that demonstrated normal trilineage hematopoiesis that occupied nearly 100% of the bone marrow, which is expected for this age. Similarly, a section of the vertebral body demonstrated cartilage surrounding pink bone with bone marrow spaces that contained normal trilineage hematopoiesis occupying nearly 100% of the bone marrow. A Kappa and Lambda ISH stain was performed that showed appropriate 2:1 Kappa to Lambda staining (Figure 6B). No gross abnormalities in the lymphatic system were noted on microscopy.

**Figure 7.**
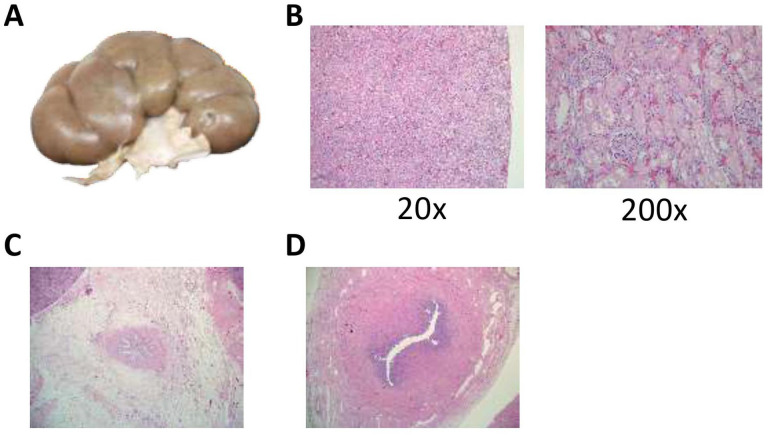
No anatomic abnormalities in the kidneys or urinary tract were identified to account for the decedent’s severe, medically refractive anasarca. (A) Representative image of the kidneys demonstrating fetal lobulation but otherwise normal in gross appearance. (B) A representative H&E micrograph of the kidney that demonstrated the appropriate maturity for the age of the decedent. There were mature glomeruli from the capsule downwards with mild autolysis present. There was also mild vascular congestion present. No atrophic tubules or chronic inflammatory infiltrate or sclerosis of the glomeruli were seen. Overall, no pathological abnormalities were identified. (H&E, 20×, 200×, as labeled) (C). A representative H&E cross section of the proximal ureter within the renal pelvis with no pathologic abnormalities or obstruction demonstrated. (H&E, 40×) (D). An H&E cross-section of the distal ureter demonstrating normal histological architecture and intact mucosa. The lumen is fully patent. (H&E, 100×).

Due to the presence of severe, medically refractory anasarca, we closely examined the genitourinary tract organs. On gross examination of the kidneys, there were no focal lesions, and the renal architecture was intact. The corticomedullary boundaries were sharply demarcated. The renal pelvises and calyces were of normal structure. There was no definite evidence of hydronephrosis or infection. However, we did note that both kidneys had fetal lobulation (Figure 7A). The combined kidney weight was 26 g, which is lower than expected for the decedent’s age (expected weight, 30 g [fifth percentile] to 57 g [95th percentile]). However, on microscopic evaluation, the kidneys had appropriate maturity for the age of the decedent, with mature glomeruli (Figure 7B). There was mild glomerular autolysis and mild vascular congestion in both kidneys. There were no atrophic tubules or chronic inflammatory infiltrate or sclerosis of the glomeruli. The renal pelvis, renal arteries, and renal veins appeared normal with no occlusions.

The ureters were straight, thin, tubular structures without focal lesions or evidence of obstruction (Figure 7C). The bladder was small and contracted and there was no evidence of hypertrophy or dilatation. The urethra was patent. There were no urethral valves or stenosis. On microscopic analysis, the distal ureters appeared normal with an inner longitudinal and an outer circular layer of smooth muscle. There was a transitional epithelium and underlying lamina propria. No obstructions or stenosis were identified (Figure 7D). The trigone of the bladder was normal with 2 patent ureters seen on cross-section. The bladder was normal on cross-section, with no edema or vascular congestion. The transitional epithelium was largely intact and appeared normal. There was normal immature female anatomy. The ovaries, fallopian tubes, uterine fundus, and cervix were normally formed; there were no gross or microscopic abnormalities.

## Discussion

The decedent had trisomy 21 with numerous other congenital abnormalities, many of which have yet to be described, particularly in a single individual. In the following paragraphs, we discuss the possible etiology of these abnormalities, their proposed effect on normal physiology, and how trisomy 21 may have led to these abnormalities based on what has been described in the literature to date.

### Trisomy 21 and associated lung and heart abnormalities

The decedent had a right-sided aortic arch with mirror imaging and an abnormal right bronchus. A right aortic arch with mirror imaging of the aorta is somewhat common in patients with tetralogy of Fallot. On the other hand, an abnormal bronchus originating from the trachea or the main bronchi is extremely rare.^10^ To our knowledge, there is only one case report of a similar, but not identical, finding of the bronchus published to date.^11^ While tracheal bronchi are rare, it has been shown that trisomy 21 is associated with an increased incidence of tracheal bronchus malformations.^1^ The particular bronchial abnormality in this patient seems to be very similar to her aorta malformation with mirror imaging of the left bronchus onto the right bronchus. The effect of this particular abnormality on the decedent’s physiology is not known. However, it is known that tracheal bronchi are associated with increased rates of pneumonia and aspiration pneumonia, which are thought to be in some part secondary to decreased ability of the affected lung to clear secretions. The presence of these rare congenital abnormalities demonstrates there were significant abnormalities in the patient’s early embryonic development, which likely contributed to the decedent’s multi-organ dysmorphia.

Mortality rates are significantly elevated for children (and adults) with trisomy 21. In fact, respiratory failure is a predictor of mortality in patients with trisomy 21.^1^ Patients with trisomy 21 have increased frequency of acute respiratory distress syndrome. Respiratory disease is the most common cause of death in persons with trisomy 21 of any age.^1^ The trachea is often smaller in those with trisomy 21, which also can have negative impact on respiration and ventilation.^1^ Therefore, it is possible that the decedent’s bronchial malformation combined with decreased trachea size contributed to the decedent’s respiratory failure and inability to be weaned off the ventilator. Moreover, previous studies have shown that individuals with trisomy 21 have decreased alveoli and have smaller lungs. For unknown reasons, individuals with trisomy 21 are 10 times more likely to develop acute lung injury.^1^ Additionally, patients with trisomy 21 have a reduced baseline lung function and they are at increased risk for the development of pulmonary hypertension (see below). The significant adhesions we found surrounding the lungs likely inhibited contraction and expansion of the lungs, further decreasing the decedent’s lung function. Overall, it is likely that all these pulmonary defects led to respiratory collapse and the inability to wean the decedent from the ventilator.

### Chylothorax

The etiology of the chylothorax was one of the clinical questions that we were asked to address in this autopsy. Chyle is a mixture of lipids that comes from the intestines after a person eats. The small intestines absorb large fatty acids, and these lipids are transported to the blood stream via the lymphatic system. The small intestine transports the lipids into lymphatic vessels that penetrate each villus. These lymphatic vessels, which are called central lacteals, drain through lymph channels within the small intestine and eventually out of the small intestine. The lymphatic vessels then drain into lymph nodes and eventually drain into larger and larger lymph node collections in the abdomen. The lymph channels eventually coalesce into the intestinal trunk located at T12 and merge with the right and left lumbar lymphatic trunks to form the cisterna chyli. The cisterna chyli then drains into the thoracic duct that traverses through the thorax to empty into the left subclavian vein. The contents of the thoracic duct then proceed to the heart to be distributed throughout the body. If there is a disruption, blockage, or any type of failure of the thoracic duct and/or one or more of its tributaries, this can result in chylothorax or, less frequently, chylous ascites.

Children with trisomy 21 have an increased risk of developing chylothorax but the mechanism causing it is not fully known.^12^ The 2 leading hypotheses are: (1) absence or incomplete formation of the thoracic duct and/or its tributaries or (2) dysfunctional lymphatic ducts, including the thoracic duct, which are not able to transport lymphatic fluid. These 2 types of abnormalities are thought to cause congenital chylothorax. Congenital chylothorax typically presents at birth or shortly after birth, once feeding is initiated. However, the structure of the thoracic duct was not abnormal in the decedent. Indeed, we were able to identify and track the thoracic duct from its origin in the abdomen to its terminus at the left subclavian vein. The duct was fully formed with no noted abnormalities or increase in size. Therefore, it is highly unlikely that the decedent’s chylothorax was due to congenital chylothorax.

The most frequent cause of chylothorax (~60% of cases) is secondary to surgery, typically thoracic surgery. During chest surgery, it is possible to inadvertently damage the thoracic duct or one of its tributaries. We did not see any evidence of damage to the thoracic duct. It is possible that a small tributary could have been damaged; however, this is typically associated with increased branching of additional tributaries into the main thoracic duct, which we did not observe in the decedent. Furthermore, the time course is not typical for a surgical injury; in these cases, chylothorax typically happens the day of surgery or a day after surgery.^13^

Another cause of chylothorax, particularly in trisomy 21 infants, is leukemia, which can cause a blockage of the thoracic duct or one of its main tributaries.^7,9^ Trisomy 21 patients have an increased risk of leukemia, particularly myeloid leukemia of Down syndrome (ML-DS) and acute lymphoblastic leukemia.^9,14,15^ Therefore, we analyzed the patient’s bone marrow and blood for leukemia. There was no evidence of leukemia in either of these 2 compartments, making a diagnosis of leukemia highly unlikely.

Interestingly, open heart surgery itself, without damage to the thoracic duct or its tributaries, can lead to chylothorax, especially in the setting of pulmonary hypertension. It is thought that this effect is due to high blood pressures being transmitted back from the lungs and heart into the venous system. These higher pressures in the venous system keep the fatty contents of the thoracic duct from being transferred efficiently into the venous system. This leads to back up of the lymphatic system and results in chylothorax and even chylous ascites.^16^ Typically, this occurs within a day of the operation as well. Overall, the most likely explanation for the decedent’s chylothorax was dramatically increased pulmonary arterial blood pressure from the decedent’s severe pulmonary hypertension with resultant systemic venous hypertension. Moreover, it is possible that there was an undetected developmental defect in the patient’s lymphatic system that made her more vulnerable for this to occur.

### Necrotizing enterocolitis, abdominal adhesions, and abnormal villi in the small intestine

The decedent had 2 episodes of necrotizing enterocolitis (NEC), which is unusual in a child that is not premature. While this can occur due to hypoperfusion and/or the administration of high doses of vasopressors, there was no evidence of shock before the development of NEC in the decedent. An additional risk factor for NEC is congenital heart disease, since it can lead to decreased profusion of tissues. While this may have contributed to the event, we also found that the decedent had extensive abdominal adhesions. While it is possible that some of these adhesions could have been from the 2 episodes of NEC, it seems unlikely that the extent of the adhesions throughout the abdomen would be from NEC alone. Moreover, we found an area of significant stenosis of the transverse colon, with proximal bowel that appeared to be somewhat ischemic, thinned and distended. Currently it is known that trisomy 21 has an increased risk of bowel atresia and stenosis for unclear reasons.^17,18^ Therefore, it is plausible that the extensive adhesions in the abdomen were related and perhaps even caused the decedent’s stenosis and/or episodes of NEC.

Interestingly, the villi in the small intestine were significantly elongated and we found a polyp that had an abnormal collagenous base, suggesting that the patient may have had some form of abnormality of collagen synthesis and/or abnormal development of the intestine. Similarly, the decedent had an abnormal amount of fibrosis around her heart and chest after surgery, which was paired with significant lung and thoracic adhesions. Altogether, these findings suggest some abnormality in collagen deposition. Several studies^19,20^ have shown that trisomy 21 patients overexpress collagen type VI and one of these studies demonstrated that collagen VI is involved in cardiac development, with overexpression predisposing a patient to AVSD.^20^ While it is possible that overexpression of collagen VI may lead to abnormal villi in the small intestine and/or increased propensity to adhesions, there is no current evidence, to our knowledge in the literature, to support this conjecture. Therefore, we do not know the underlying cause of these findings and whether they are clinically significant. Moreover, it is not completely clear if the abnormally elongated villi are related to the significant of adhesions and/or the multiple instances of NEC in the decedent.

### Necrosis of the pancreas

A surprising finding was that the head of the pancreas, containing the biliary duct and pancreatic duct, was necrotic. This necrosis was likely very acute and close to the patient’s death since there was minimal biliary congestion in the liver. Indeed, if the biliary tract were completely blocked, even for a day, there would likely be significant congestion of the liver with bile. Moreover, the alkaline phosphatase near the decedent’s death was 112 IU/L, which is below the normal level. Alkaline phosphatase is a marker for biliary obstruction, and this would have likely increased dramatically if there were occlusion for more than 24 hours. Therefore, based on this evidence, it is likely that the necrosis of the pancreas occurred soon before the patient’s death.

### Trisomy 21 and pulmonary hypertension

Infants with trisomy 21 have a high rate of pulmonary artery hypertension of the newborn.^1^ It is thought that the combination of upper tract respiratory malformations, a diminished number of alveoli, a thinner media of pulmonary arterioles, impaired endothelial function, alveolar capillary dysplasia, and hypoxia and hypercapnia may collectively promote and sustain pulmonary hypertension in trisomy 21 patients.^1,3^ Moreover, it has been shown that in trisomy 21 patients, pulmonary arterial hypertension may persist even after correction of the shunting of blood.^1^

The decedent was found to have extensive thickening of her pulmonary arterioles and arteries. There was also calcium deposition in many of the arteries and arterioles, which is indicative of chronic pulmonary hypertension. However, the level of calcification was exceedingly high and this level of calcification is not typically seen in infants or children with pulmonary hypertension. Instead, the amount of calcification that was observed is typically seen in adults with long-standing (ie, many years) chronic pulmonary hypertension. Therefore, it seems that the decedent was exceptionally prone to developing severe pulmonary hypertension, even in the short duration that she experienced increased pulmonary arterial pressures.

### Inability to be weaned from the ventilator

In addition to significant pulmonary hypertension, there was extensive congestion of the lungs bilaterally. Trisomy 21 patients are known to have fewer alveoli compared to unaffected individuals and to have less developed lungs compared to children of the same age.^5,6^ With significant congestion of the decedent’s lung this led to decreased oxygen exchange. Moreover, there already was a moderate amount of fibrosis present within the lungs that would also lead to decreased oxygen and carbon dioxide exchange. Finally, there was extensive adhesions seen both macroscopically and microscopically on the surface of the lung that would impede the expansion and contraction of the lungs. With all these findings, it is evident that these significantly contributed to the decedent’s inability to be weaned from the ventilator.

### Anasarca

Another clinical question that we considered was the patient’s underlying anasarca. Edema and anasarca typically have 4 major causes: (1) elevation of capillary hydraulic pressure, (2) increased capillary permeability, (3) lymphatic obstruction, and (4) hypoalbuminemia. Elevation of capillary hydraulic pressure in an infant is usually from heart failure, kidney disease, or early cirrhosis. The patient did have kidneys that were small for her age, but the decedent was also small (underweight and decreased height) for her age. On microscopic evaluation, the kidneys had a normal appearance and were appropriately mature for the decedent’s age. There was no evidence of nephritis or nephrotic syndrome and no indication of acute or chronic kidney disease. Her liver on microscopic evaluation appeared nearly normal with only mild biliary stasis, as discussed previously. Nevertheless, it is possible that while the patient’s kidneys appeared normal microscopically there was an underlying defect in a protein(s) that did not allow them to function properly. She had no indication of intrinsic liver damage or damage secondary to hepatic congestion from heart failure. Her portal vein was completely patent. Her heart, while enlarged, had a normal microscopic appearance and had no signs of ischemic damage to either ventricle. On review of her echocardiogram and cardiac catheterization, she had good heart function and a regular ejection fraction, indicating that her heart was functioning well and there was no backup of blood from the right side of her heart into her venous system (which causes edema in heart failure).

Increased capillary permeability typically is caused by sepsis, burns, or trauma. The patient was not burned. She did not have sepsis based on her negative blood cultures. The patient did have trauma from her recent operations, but these operations are highly unlikely to have induced the type of full body edema that was seen in this patient. Moreover, the edema that can be seen after surgery is typically responsive to diuretics. Lymphatic obstruction was unlikely in this patient as we were able to clearly delineate the thoracic duct and rule out an obstruction. Even if the patient had a blocked tributary of the thoracic duct, this should not cause the widespread edema that was seen in the decedent. Malignancy, as discussed before, can occlude the thoracic duct and lead to edema; however, there was no indication that the decedent had a malignancy.

Hypoalbuminemia is an alternative potential cause. This can be due to severe kidney dysfunction, liver failure, or to severe malnutrition. However, there is no indication of kidney failure or liver failure, based on microscopic and laboratory values. The patient’s last albumin level was 3.5, which is low, but in the normal range, and is not indicative of severe malnutrition. Therefore, it does not seem that any of these mechanisms caused the patient to be severely edematous.

Systemic capillary leak syndrome is an rare cause of anasarca that was first described in 1960.^21^ The syndrome is a diagnosis of exclusion in patients with anasarca and the triad of hypotension, hemoconcentration, and hypoalbuminemia.^21,22^ In adults, but not in children, the vast majority of patients (~80%) have monoclonal gammopathy of undetermined significance.^22^ The majority of these patients also tend to have waxing and waning symptoms (if the first episode does not lead to death). The decedent did have consistently low albumin levels (average 2.87 g/dL; range 3.8-1.9 g/dL [reference range 3.5-5.0 g/dL]) and protein levels (4.63 g/dL; range 6.0-3.4 g/dL [reference range 6.5-8.3 g/dL]) throughout her hospitalization. She was also consistently hypotensive. The patient had periods of hemoconcentration, but other times she was either within or below the normal range (average 36.9%; range 62.6%-25.9% [reference range 28.0-42.0]); however, the periods of her most severe hemoconcentration do seem to correlate with intervals when she developed anasarca. Therefore, it is possible the decedent’s anasarca was caused by or at least had contribution from the presence of systemic capillary leak syndrome. Finally, another possible explanation for her underlying anasarca was her severe pulmonary hypertension causing backup of fluid from her venous and/or lymphatic system. This could explain why she had both anasarca and chylous pleural effusions.

## Conclusion

The decedent had multiple, coexisting rare congenital abnormalities that negatively affected the decedent’s physiology. These abnormalities together combined to decrease the decedent’s ability to function without medical support. Some of these abnormalities are not well described (or described at all) in the literature about trisomy 21 and likely affected the decedent’s physiology in unknown ways. Overall, the main driver of the decedent’s demise was pulmonary arteriolar changes leading to pulmonary hypertension that was significantly out of proportion to what would be expected in a patient with tetralogy of Fallot and severe pulmonary stenosis. Indeed, one would expect the pulmonary arteries not to be exposed to high flow and systemic blood pressure with the level of pulmonary stenosis present. Thus, the severity of pulmonary arteriolar changes observed in the lung tissue was unexpected. This unrecognized, severe early and accelerated pulmonary vascular obstructive disease contributed to the development of pulmonary hypertension and significantly contributed to the demise of the patient. Additionally, the propensity for adhesion formation throughout her body directly affected her gastrointestinal system leading to recurrent episodes of NEC and ileus and exacerbated her lung disease causing restrictive lung physiology. In turn, this problem worsened her malnutrition and her respiratory status, respectively. Finally, all these pathophysiologic abnormalities resulted in increased systemic venous pressures causing decreased lymphatic flow paired with recurrent loss of plasma proteins and immunoglobins from the chylous effusion. These, plus possibly co-existing capillary leak syndrome, resulted in the severe, medically-refractive anasarca.

Overall, one can image the amount of dysfunction from trisomy 21 as a bell curve, with nearly normal function at one extreme and near-to-absolute incompatibility with life at the other extreme. Unfortunately, it appears that the decedent fell on the side of the bell curve that is nearly incompatible with life, and beyond correction by current medical or surgical interventions. As critical care technologies and surgical care continue to advance, with the ability to sustain life in neonates and infants that, in the past, would have died immediately after birth, it is likely that severe trisomy 21 phenotypes, such as in this case may become more common in tertiary and quaternary centers. Thus, reporting the clinical course and autopsy findings in this severe case of trisomy 21 may help guide care and eventually lead to better outcomes for these patients.
